# Insomnia, Alcohol Consumption and ADHD Symptoms in Adults

**DOI:** 10.3389/fpsyg.2020.01150

**Published:** 2020-05-27

**Authors:** Astri J. Lundervold, Daniel A. Jensen, Jan Haavik

**Affiliations:** ^1^Department of Biological and Medical Psychology, University of Bergen, Bergen, Norway; ^2^Department of Biomedicine, University of Bergen, Bergen, Norway; ^3^Division of Psychiatry, Haukeland University Hospital, Bergen, Norway

**Keywords:** ADHD, Insomnia, alcohol, depression, adults

## Abstract

**Introduction:**

Substance use disorders and insomnia are common in the general population, and particularly among adults with attention-deficit/hyperactivity disorder (ADHD). Here we investigated the relationship between insomnia, alcohol consumption and ADHD symptoms.

**Methods:**

Adults with an ADHD diagnosis (*n* = 235, 41.3% males) and controls (*n* = 184, 38% males) completed a questionnaire assessing insomnia (Bergen Insomnia Scale), alcohol consumption (Alcohol Use Disorders Identification Test), and current ADHD symptoms (Adult ADHD Self-report Scale). The majority of the sample (95%) gave additional information about childhood ADHD symptoms (Wender Utah Rating Scale), and information about lifetime occurrence of an internalizing disorder was included as part of background information.

**Results:**

Compared to controls, the ADHD group reported a higher frequency of insomnia, a higher quantity of consumed alcohol and a higher frequency of internalizing disorders. Current and childhood ADHD symptoms were more severe in those with than without insomnia. Scores on ADHD symptom scales were explained by the presence of insomnia and internalizing disorders, while the contribution from alcohol consumption was restricted to the control group.

**Discussion:**

The high functional impact of insomnia, alcohol misuse and internalizing disorders is well known. The present study contributed by focusing on their relations to ADHD symptoms, and by showing that strong relations were not restricted to adults with a clinical ADHD diagnosis. By this, the results put a critical light on a categorical delineation between adults with an ADHD diagnosis and population selected controls, and call for further studies including dimensional metrics of ADHD symptoms and co-occurring problems.

## Introduction

Attention-deficit/hyperactivity disorder (ADHD) is a neurodevelopmental disorder, characterized by symptoms of inattention and/or hyperactivity/impulsivity that are persistent across situations and time (American Psychiatric Association [APA], 2013). ADHD in adulthood, with an estimated prevalence of 2–3% (Faraone and Biederman, 2005), is associated with many daily-life challenges, including problems related to poor achievements at school and work (Daley and Sayal, 2006; Küpper et al., 2012), high rates of comorbid mental disorders (Haavik et al., 2010; Solberg et al., 2019), somatic diseases (Instanes et al., 2018), accidents with injuries and even early mortality (Franke et al., 2018). The present study investigates two co-existing problems that are associated with negative functional outcome in adults with ADHD as well as among adults in the general population: insomnia and a high quantum of consumed alcohol.

Insomnia is characterized by sleep loss due to problems initiating and maintaining sleep and/or early-morning awakening, with an inability to return to sleep. Insomnia is thus a sleep disorder with potentially high impact on the lives of those afflicted (see e.g., Sivertsen et al., 2009). Insomnia is common, with a population prevalence around 30% (Theorell-Haglow et al., 2018), with adults with ADHD having an up to fivefold higher risk of insomnia compared to population controls of similar age (Brevik et al., 2017). Brevik et al. (2017) showed that 31% of the variance in reports on an insomnia symptom scale could be explained by current ADHD symptoms, supporting a close relation between severity levels of sleep problems and ADHD symptoms (Schredl et al., 2007). Other studies have suggested that the close association between insomnia and ADHD symptoms at least partially is explained by overlapping features. This implies that individuals with ADHD may have high rates of comorbid insomnia and that insomnia patients may have ADHD, but also makes it reasonable to expect that adults with sleep problems in the general population may display symptoms typically associated with ADHD. Insomnia may thus be misdiagnosed as ADHD and vice versa (e.g., Owens, 2005; Gregory et al., 2017). Furthermore, sleep disorders and ADHD symptoms share several cognitive and emotional features (Musser and Raiker, 2019), features that are also shared with diagnostic categories such as depression (Baglioni et al., 2011; Lundervold et al., 2016) and anxiety (Bragantini et al., 2019). The co-existence of sleep problems and ADHD is also well established in childhood (e.g., Yoon et al., 2012) and adolescence (e.g., Hysing et al., 2016), but less is known about trajectories of sleep problems from childhood to adulthood (Cortese, 2015). Although the severity of sleep problems and ADHD symptoms are shown to be predictors of persistent sleep problems, Gregory et al.’s (2017) longitudinal twin study concluded that the risk for sleep problems in adulthood was restricted to those with an ADHD diagnosis persisting from childhood. This result emphasizes the importance of including information about childhood symptoms in studies of sleep problems in adults with ADHD, and to be ready to explore associations between insomnia and ADHD symptoms also in individuals without a formal ADHD diagnosis.

Substance use disorders (SUDs) are also common in adults with ADHD (Kessler et al., 2006). Like insomnia, substance abuse shares several features with ADHD symptoms. This feature sharing can be illustrated by results from an international study showing that around 40% of patients with a SUD screened positive for an ADHD diagnosis, with an even higher number in a Norwegian sample (Glind et al., 2013). Furthermore, a study by Hagen et al. (2017) showed a substantial reduction in ADHD symptoms in participants with SUD who remained abstinent for 1 year. Although the severity of co-occurrent drug use disorder in adults with ADHD should be emphasized, the severity of problematic alcohol certainly needs awareness. Alcohol use disorder (AUD) is not only the most common substance abuse disorder in the population (Kessler et al., 2006; Kabashi et al., 2019), but also a disorder with a high prevalence in adults screening positive for an ADHD diagnosis (Glind et al., 2014). The high frequency of anxiety and depression among adults with a harmful alcohol use add to the risk of negative functional outcome for those afflicted (Kabashi et al., 2019). From these studies, it should not be a surprise that a high level of alcohol consumption affects the lives of many adults with ADHD (Kronenberg et al., 2014). Even the presence of ADHD symptoms without having a clinical ADHD diagnosis is shown to increase the risk for problems related to alcohol consumption (DeAlwis et al., 2014; Daurio et al., 2018). The importance of ADHD symptoms is further underscored by studies showing that childhood ADHD is a strong predictor of AUD in adulthood (Kuperman et al., 2001) and that a high polygenic risk for ADHD is associated with increased risk of SUD (Wimberley et al., 2019). Research has shown mixed findings regarding cut-off values indicating AUD, and different values have been presented for men and women and for specific countries (see World Health Organization [WHO], 2000; DeMartini and Carey, 2012; Kabashi et al., 2019). Other studies have defined problematic alcohol use more directly from items assessing frequency of drinking and/or consumption quantity. Although some studies have reported a high phenotypic correlation between the two (Sudlow et al., 2015), Marees et al. (2020) have recently suggested that these two metrics have opposing consequences on several aspects of mental health. Of interest to the present study, Marees et al. (2020) showed that consumption quantity is a stronger risk factor in adults with ADHD than the frequency of drinking behavior.

The association between problematic alcohol use and insomnia is well established (e.g., Ebrahim et al., 2013; Koob and Colrain, 2020). Insomnia may both precede a harmful alcohol use, and insomnia may also be persistent during months of abstinence (Brower, 2015). The presence of insomnia and problems related to alcohol consumption along the full spectrum of ADHD symptom severity is, however, still not fully understood. This calls for studies including a sample that is not restricted to adults with an ADHD diagnosis. This motivated us to investigate the relationship between the diagnostic category of insomnia, the severity, frequency and quantity of alcohol consumption and the full range of ADHD symptoms in a sample of adults with an ADHD diagnosis and a sample of adults recruited from the population. From previous studies we expected the ADHD group to show a higher frequency of insomnia and a higher quantity of alcohol consumption than the control group, and that both measures of insomnia and alcohol consumption would be associated with severity level of current and childhood ADHD symptoms. Finally, we expected that these relationships would be found both in the sample of adults with a clinical ADHD diagnosis and in the population-based sample.

## Methods

### Sample

The included adults were part of a larger cross-sectional study^1^ where most of the participants in the ADHD group were recruited from a national registry of adults diagnosed in Norway from 1997 to May 2005. The diagnostic assessment was made by expert committees according to the ICD-10 research criteria (World Health Organization [WHO], 2003), with allowance for using the DSM-IV criteria for an ADHD diagnosis (American Psychiatric Association [APA], 2000). The participants in the control group were randomly recruited from the Medical Birth Registry of Norway, a registry including all individuals born in Norway after January 1st, 1967. See Halleland et al. (2012) for more information about the sample. The study was approved by the Norwegian Regional Committee for Medical and Health Research Ethics, RECWest [IRB #3 (FWA00009490, IRB00001872)]. The present study included all adults who responded on the relevant items of a questionnaire assessing current ADHD symptoms, insomnia and alcohol use. Reports of childhood symptoms and lifetime internalizing disorder were obtained from more than 90% of the included adults.

### Materials and Methods

#### Insomnia

Insomnia was assessed by self-reports on the Bergen Insomnia Scale (BIS) (Pallesen et al., 2008), including six items, each evaluated on a scale from zero to seven. The first four items assess sleep impairment (criteria A of the DSM-IV) and the last two items refer to daytime sleepiness/tiredness that has affected participation at school or work, and dissatisfaction with sleep, respectively (criteria B of the DSM-IV). *Insomnia* was defined when reporting ≥3 days per week on at least one of the A-items and ≥3 days per week on at least one B item according to the criteria given in the DSM-IV (American Psychiatric Association [APA], 2000).

#### Alcohol Consumption

Alcohol consumption was assessed by self-reports on the Alcohol Use Disorder Identification Test (AUDIT; Babor et al., 2001). The scale includes 10 items, where the first eight give information about the extent or severity of drinking behavior (answered in terms of standard drinks and number of days drinking), and the last two about others being injured or concerned about their drinking behavior. Research has shown mixed findings regarding cut-off values indicating AUD, and different values have been presented for men and women and from specific countries (see World Health Organization [WHO], 2000; DeMartini and Carey, 2012; Kabashi et al., 2019). In the present study, where we are interested in associations along the full dimension of ADHD symptoms, we included a dimensional measure of alcohol consumption. This measure was defined as the sum score across the first eight AUDIT items, where higher scores indicate a more severe problem related to alcohol consumption. Inspired by the results presented by Marees et al. (2020), we added detail information about the frequency of drinking behavior (AUDIT item # 1) and the quantum of alcohol consumption on a typical day of drinking (AUDIT item # 2).

#### Childhood ADHD Symptoms

The 25-item version of WURS (Ward et al., 1993) was used to assess ADHD related behavior in childhood. According to the instructions, the participants rated each item on a 0 (“not at all or very slightly”) to 4 (“very much”) scale based on their recall of ADHD related childhood behavior. The items are chosen from the original 61 item version for their ability to discriminate between ADHD and controls, and the long-term reliability of WURS-25 was recently supported (Lundervold et al., 2019). The sum score across the 25 items was used as a severity measure of childhood symptoms in the present study.

#### Current ADHD Symptoms

Current ADHD symptoms were assessed by the 18-item version of ASRS (Kessler et al., 2007) addressing the DSM criteria for an ADHD diagnosis (American Psychiatric Association [APA], 2013). The participants were asked to rate their ADHD related behavior in the past 6 months on a scale from 0 (“never”) to 4 (“very often”). The sum score across the 18 items was used as a severity measure of ADHD symptoms in the present study.

#### Background Variables

The majority of the participants provided information about gender, age, education, work, if they had an ADHD diagnosis as a child and if they ever had experienced significant episodes of depression or anxiety. The latter was included as a measure of lifetime occurrence of an internalizing disorder, defined from a “yes” response on the following question: “Have you ever experienced significant anxiety or depression?”

### Statistical Analyses

The SPSS software, version 25, was used for all statistical analyses. A set of explorative analyses included independent samples *t*-tests (with Levene test) and Chi-square tests when appropriate. The associations between the severity measure of alcohol consumption and the scores on the two symptom scales were included in a bivariate correlation analysis. Finally, a set of regression analyses were computed with each of the two ADHD symptom scales as the outcome variable. A preparatory step was included to identify background variables that should be included as control variables. Then a first set of regression analyses included only insomnia or alcohol consumption as independent variables, followed by a set of analyses including these two variables together with the control variables selected in the preparatory step. The results were calculated for the full sample and for each of the two groups.

## Results

### Description of the Sample

#### Background Information and the Presence of Insomnia

The ADHD (*n* = 235) and the control group (*n* = 184) were not significantly different regarding gender and age, but the ADHD group included fewer participants with the highest education than the control group and a lower percentage in active work participation (Table 1). A total of 186 adults reported lifetime occurrence of an internalizing disorder and 211 adults reported insomnia, with a significantly higher proportion in the ADHD group than in the controls. The number of adults with internalizing disorders was significantly higher in those with (*n* = 125 adults, *n* = 109 from the ADHD group) than without insomnia (*n* = 55). In the ADHD group, the proportion of participants reporting insomnia and internalizing disorders were similar among adults who reported that they had been diagnosed with ADHD during childhood and participants who had been diagnosed as adults, and the symptom scores were not significantly different between the two ADHD subgroups (*p* > 0.01).

**TABLE 1:** Descriptions of the two samples.

	**ADHD group (*n* = 235)**	**Control group (*n* = 184)**
Female/males (n/n)	138/97	114/70
Age [mean (SD)]	37.91 (11.2)	36.55 (8.2)
University/high school/lower (%)	34.3/47.3/18.4%	77.8/17.2/5%***
Work (%)	40.2%, *n* = 88, 45 females	88.4%, *n* = 152***, 92 females
Internalizing disorder (%)	64.7%, *n* = 152, 98 females	17.4%, *n* = 32***, 24 females
Insomnia (*n*/%)	67.2%, *n* = 158, 99 females	28.8%, *n* = 53***, 37 females
AUDIT sum score, eight items:	13.59 (4.79), *n* = 235	12.32 (2.69)**, *n* = 184
*t*-test: ADHD vs. controls		*t*(381.42) = 3.44, *p* = 0.001, *d* = 32
Alcohol severity no insomnia	12.81 (3.79), *n* = 77	12.13 (2,97), *n* = 131
Severity with insomnia	13.97 (5.17), *n* = 158	12.77 (2.97), *n* = 53
*t*-test: with/without insomnia	*t*(233) = 1.76, *p* = 0.080	*t*(182) = 1.38, *p* = 0.142
ASRS score	42.18 (12.50), *n* = 235	21.77 (9.78)***, *n* = 184
*t*-test: ADHD vs. Controls		*t*(417) = 18.76, *p* < 0.001, *d* = 1.79
WURS score	51.80 (17.66), *n* = 218	16.59 (15.47)***, *n* = 182
*t*-test: ADHD vs. controls		*t*(397.1) = 21.25, *p* < 0.001, *d* = 2.10
ASRS score no insomnia	36.57 (12.54), *n* = 77	20.13 (8.60)***, *n* = 131
ASRS score with insomnia	44.91 (11.57), *n* = 158	25.81 (11.31)***, *n* = 53
*t*-test: with/without insomnia	*t*(140.45) = 3.30, *p* = 0.001, *d* = 0.70	*t*(77.54) = 3.29, *p* = 0.001, *d* = 60
WURS score no insomnia	46.15 (17.81), *n* = 71	13.75 (12.84)***, *n* = 129
WURS score with insomnia	54.16(16.99), *n* = 147	23.49 (18.95)***, *n* = 53
*t*-test: with/without insomnia	*t*(132.72) = 9.64, *p* < 0.001, *d* = 0.46	*t*(72.44) = 3.43, *p* = 0.001, *d* = 0.66

Alcohol severity, sum score across the first eight AUDIT items; *** *p* < 0.001; ** *p* < 0.005; * *p* < 0.05. The *d*-scores are corrected for unequal sample sizes.

#### Alcohol Consumption

Table 1 shows that the AUDIT sum score across the first eight items, used to define severity of alcohol consumption, was significantly higher in the ADHD group than in the controls, with a lower score in females than males in both the ADHD (*p* = 0.002) and the control group (*p* = 0.006). The differences were non-significant when comparing those with and without insomnia and when comparing alcohol consumption in those with and without internalizing disorders within each of the two groups. The differences were, however, statistically significant when the full sample was included in the analysis, both for insomnia [*t*(361.04) = 3.30, *p* = 0.001] and internalizing disorders [*t*(287.26) = 2.65, *p* = 0.008]. The alcohol use was also more severe in the 27 adults with ADHD who reported a childhood diagnosis [15.59 (5.89)] than in adults with ADHD without such a diagnosis [13.30 (4.59), *t*(230) = 2.35, *p* = 0.019].

The difference between the ADHD and the control group was much higher for alcohol consumption quantity than for frequency of use, with 42.1% of the ADHD group and 21.1% of the control group drinking at least 5–6 units, with 8.5% (*n* = 20) in the ADHD and 1.6% (*n* = 3) in the control group reporting drinking 10 or more units every time of drinking. The corresponding percentages for a frequency of at least 2–3 times a week were 57.2% for the ADHD group and 69% for the control group. Among those with insomnia, 46.9% in the ADHD and 24.6% in the control group reported drinking at least 5–6 units, with 10.8% (*n* = 17) in the ADHD and 3.8% (*n* = 2) in the control group reporting drinking 10 or more units every time of drinking. Among the 109 adults in the ADHD group with both insomnia and internalizing disorder, 11% reported alcohol consumption at the highest level.

### ADHD Symptoms, Insomnia and Alcohol Consumption

**FIGURE 1:**
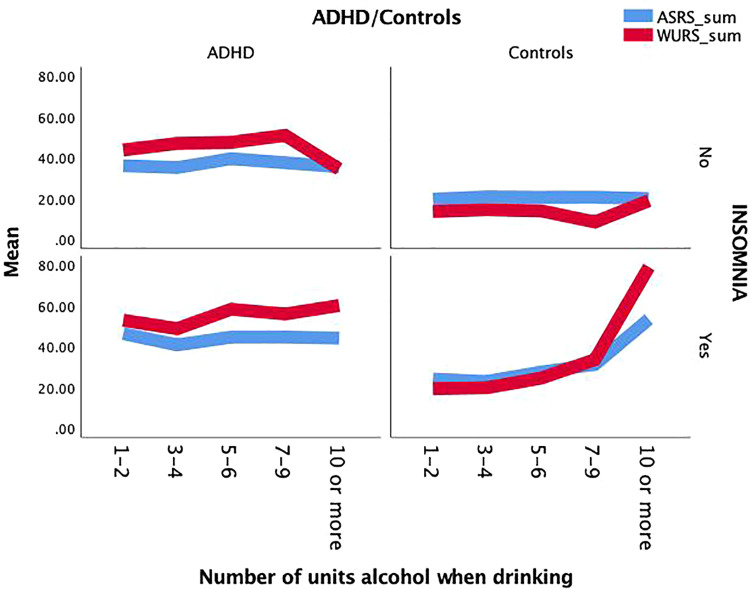
The sum score of ASRS and WURS as a function of group, the presence of insomnia and the number of consumed alcohol units on a typical day of drinking in the ADHD and the control group.

The range of scores on the ASRS and the WURS were from 4–70 and 3–97, respectively. The ranges were wide in both the ADHD (8–70 and 12–97) and the control group (4–56 and 0–87), with significantly higher mean scores in the ADHD group on both scales and with significantly higher scores in adults with than without insomnia (Table 1). Statistically significant bivariate correlations were found between the severity score of alcohol consumption and the ASRS (*r* = 0.347, *p* < 0.001) and the WURS scores (*r* = 0.384, *p* < 0.001) in the control group, and for none of the scales in the ADHD group (*r* = 0.002, and *r* = 128, respectively). Figure 1 illustrates that the score on the two symptom scales were dependent on the number of units consumed each time of drinking, with the most convincing rise on the WURS score for the few participants in the control group (*n* = 3) with a very high quantity of alcohol consumption.

#### Contributions From Insomnia and Alcohol Consumption on Reports of ADHD Symptoms

The preparatory regression analysis, computed to select control variables to be included in the following analyses, identified statistically significant contributions from education and internalizing disorders. The first step of the analysis, including either insomnia (a) or severity of alcohol consumption (b) as the independent variable, showed that each variable contributed significantly to explain the two symptom scores, with insomnia contributing with as much as 20% explained variance in the full sample (Table 2). When education and internalizing disorders were entered together with insomnia and severity of alcohol consumption (c), internalizing disorders as well as insomnia contributed with statistically significant effects on both symptom scales and in both groups, while the impact from education was restricted to the WURS scale. A statistically significant contribution from our measure of severity of alcohol consumption was only found in the control group.

**TABLE 2:** Linear regression analysis with ADHD symptom scores as outcome variables.

	**ASRS, *n* = 419**			**WURS, *n* = 400**		
**Predictors**	**β**	**R^2^**	**ΔR^2^**	**β**	**R^2^**	**ΔR^2^**
**All: Adhd + controls**						
(a) Insomnia	0.457***	0.207	0.208***	0.435***	0.187	0.189***
(b) Alcohol severity	0.182***	0.026	0.029***	0.321***	0.060	0.062***
(c) Education	0.171***	0.134	0.136***	0.307	0.250	0.252***
Internalizing	0.328***	0.285	0.153***	0.376	0.424	0.175***
Insomnia	0.286***	0.354	0.070***	0.201	0.461	0.037***
Alcohol severity	0.064	0.356	0.004	0.134	0.476	0.017**
**Adhd**						
(a) Insomnia	0.314***	0.095	0.098***	0.223***	0.045	0.050**
(b) Alcohol severity	0.002	0.004	0.001	0.128	0.012	0.016*
(c) Education	0.006	0.000	0.005	0.310***	120	0.124***
Internalizing	0.180*	0.042	0.046*	0.271***	0.209	0.085***
Insomnia	0.292***	0.118	0.080***	0.130*	0.227.	0.017*
Alcohol severity	0.032	0.114	0.001	0.086	0.234	0.007
**Controls**						
(a) Insomnia	0.264***	0.065	0.070***	0.287***	0.077	0.082***
(b) Alcohol severity	0.347***	0.115	0.120***	0.384***	0.143	0.147***
(c) Education	0.139	0.080	0.085***	0.144	0.097	0.102***
Internalizing	0.192*	0.117	0.042**	0.253	0.162	0.070***
Insomnia	0.153*	0.141	0.028*	0.156	0.188	0.030*
Alcohol severity	0.292***	0.217	0.079***	0.324	0.283	0.098***

Alcohol severity, sum score across the first eight AUDIT items; internalizing, self-reported lifetime occurrence of depression or anxiety; β, standardized coefficient; R^2^, adjusted R-square; ΔR^2^, the unique R-square change for the given variable. *** *p* < 0.001; ** *p* < 0.005; * *p* < 0.05.

## Discussion

The present study showed a higher frequency of insomnia and a higher frequency and quantity of alcohol consumption in adults with ADHD than in adults selected from the general population. Participants with insomnia reported higher current (ASRS) and childhood (WURS) ADHD symptoms scores and a higher level of alcohol consumption than adults without insomnia. The presence of insomnia, the severity level of alcohol consumption and number of alcohol units consumed each time of drinking were associated with severity level of ADHD symptoms. When controlling for demographic variables and insomnia in a linear regression analysis, the severity of alcohol consumption contributed significantly to the ADHD symptom scores only in the control group, while insomnia and lifetime occurrence of internalizing disorder retained significant contributions in both groups.

The high prevalence of insomnia and alcohol use in the ADHD group is expected from results in previous studies, showing a higher risk of both insomnia (Brevik et al., 2017) and alcohol use (Glind et al., 2014) in adults with ADHD than in adults from the general population. Furthermore, we confirmed a strong association between insomnia and internalizing disorders (Baglioni et al., 2011; Bragantini et al., 2019). However, the relationships previously reported between problematic alcohol consumption and insomnia (Ebrahim et al., 2013; Koob and Colrain, 2020) and between internalizing problems and an AUD (Marmorstein, 2010; Jo and Won, 2018; Kabashi et al., 2019) were only confirmed in our full sample. Participants with ADHD did, however, consume more units of alcohol on a typical drinking day than the control group from the general population. This high quantity of alcohol consumption in adults with an ADHD diagnosis and co-occurring insomnia should be emphasized. About 45% of those adults reported that they consumed at least 5–6 units of alcohol on a typical day of drinking, with almost 11% consuming 10 or more units. Regarding frequency of drinking behavior, the metrics were much more similar between the two groups. By this, the results supported the importance of consumption quantity, as emphasized by Marees et al. (2020) and studies showing the harmful effect of binge drinking (Kabashi et al., 2019). Marees et al. (2020) did also show that low socioeconomic status (SES) was related to high quantity of consumption and high SES to high frequency of drinking behavior. Although detailed information about SES was not included in the present study, we confirmed a relationship between level of education (which are closely related to SES) and drinking behavior (Russell et al., 2016). Due to the close relationship between SES and negative consequences of alcohol use (Skogen et al., 2019), future studies should investigate the importance of SES on the relation between ADHD symptoms and problems associated with insomnia and alcohol consumption.

The results from the present study support arguments for a dimensional view on ADHD symptoms (Faraone and Biederman, 2016) and for including information about both current and childhood symptoms when evaluating adults presenting problems related to alcohol use and sleep problems (Daurio et al., 2018). We found that a dimensional view fitted well with characteristics of the present sample, containing a population-based control sample and an ADHD sample where only a minority reported a childhood diagnosis. Therefore, our sample may both have included controls with a subthreshold childhood ADHD diagnosis and adult onset cases within the ADHD group (Moffitt et al., 2015; Schiavone et al., 2019). By this, reports from our sample covered almost the whole range of scores across the ADHD symptom scales, and gave results opposing a strict boarder between reports from the two groups. Overlapping reports from the adults in the ADHD groups and controls suggest that the presence of ADHD symptoms should always be taken into account when targeting treatment programs to adults with sleep problems like insomnia and/or a high quantity of alcohol consumption (see Jernelöv et al., 2019), and that co-occurring sleep problems and alcohol related problems should be assessed even in adults with current or childhood ADHD symptoms at a diagnostically subthreshold level.

The present study confirmed previous studies showing a female predominance both regarding reports of ADHD symptoms (Vildalen et al., 2019), insomnia (Theorell-Haglow et al., 2018), sleep problems (Arnedt et al., 2011) and the presence of internalizing disorders (Ottosen et al., 2019; Solberg et al., 2019). Regarding alcohol consumption, males reported higher AUDIT scores than females. This may be explained by a self-report-bias due to lower acceptance of alcohol use in females than males, but our findings are in accordance with results showing that women are more often lifetime abstainers and drink less than men do (see Erol and Karpyak, 2015 for a review). Future studies investigating interactions between biological sex and gender-related factors are, however, needed to identify and treat risk factors for AUDs in both men and women.

### Strengths and Limitations

The main strength of the present study is the inclusion of a clinically validated patient sample and a control sample randomly recruited from the Norwegian population without any formal exclusion criteria. We believe that this strengthens the validity and clinical utility of our findings. As already mentioned, there is a potential for some undiagnosed cases of ADHD being included in our control group and that adult onset cases may have different characteristics than cases identified in childhood. All cases and controls reported past and present ADHD symptom scores using the ASRS and WURS to screen for ADHD symptoms. Based on reported cut-off scores on these scales and an around 2% estimated prevalence rate of ADHD in the adult population (Solberg et al., 2019), we believe that a possible contribution from a small number of individuals with ADHD symptoms in the control group cannot explain the main findings of the present study. We rather suggest that the inclusion of the two samples of adults was a strength by enabling us to study associations along the full spectrum of ADHD related symptoms.

The results should, however, be viewed in light of several limitations. First of all, self-reports may have biased the results. Reports on alcohol use may for example be under-reported while mental health complaints like insomnia may be overrated. Furthermore, recall bias may have influenced the retrospective reports of childhood behavior on the WURS (Miller et al., 2010; Lundervold et al., 2019). The ASRS score may on the other hand be more affected by short term confounders, such as time of day (Franke et al., 2018), affective fluctuations (Lundervold et al., 2011), other sleep problems (Bjorvatn et al., 2017) and other comorbid disorders (Haavik et al., 2010) that are known to affect the severity level of symptom reports in adults with ADHD. Our measure of alcohol consumption may also be considered as a limitation. We included a dimensional measure of alcohol consumption defined from the sum of the first eight AUDIT items. Surprisingly, the contribution of this measure to explain the ADHD symptom scores was non-significant, suggesting that the adults may have used a different, and probably more reluctant, response-set when reporting symptoms on the AUDIT than on the ADHD rating scales. Furthermore, the response-categories in the present study were different from the ones used in studies providing prevalence rates of risky alcohol consumption (e.g., Conigrave et al., 1995; DeMartini and Carey, 2012; Kabashi et al., 2019). By this, we were not able to provide prevalence data on risky alcohol consumption that are comparable to results from previous studies. On the other hand, we gained informative information about the association between dimensional measures of alcohol consumption and ADHD symptoms. Furthermore, the add-on information about frequency and quantum of alcohol consumption gave us metrics that were comparable to the ones presented by Marees et al. (2020). Our definition of insomnia from self-reports may also be criticized, and it is important to emphasize that subjective measures of insomnia not necessarily correlate with objective measures of sleep quality (Philipsen et al., 2005; Schredl et al., 2007). The frequency of insomnia shown in the control sample (28.8%) was, however, close to the 30% reported in previous population-based studies (Theorell-Haglow et al., 2018). With the 67.2% occurrence in the ADHD group shown in the present study we also assume that our definition demonstrated the strong link between ADHD and insomnia.

## Conclusion

The present study indicated a strong impact of insomnia and the presence of lifetime internalizing disorders on core symptoms of ADHD, but the direction of the causality could not be stated from the available data. Although adults with an ADHD diagnosis showed signs of a higher quantum of alcohol consumption than controls, its impact on level of ADHD symptoms was restricted to the control group when insomnia and lifetime internalizing disorders were controlled. The present study should therefore inspire further studies on the role of co-existing problems along the full dimension of ADHD symptoms. Information from such studies are expected to improve our assessment and ability to target treatment programs for adults disturbed by symptoms associated with an ADHD diagnosis as well as in adults with problems like insomnia, AUD, depression and anxiety. The stronger association between alcohol consumption and ADHD related symptoms in the control group put a critical light on a categorical delineation between adults with a clinical ADHD diagnosis and controls.

## Data Availability Statement

The data are not publicly available due to restrictions in the approval given by the Regional Committees for Medical and Health Research Ethics in Norway. The data that support the findings of this study are available on request from the corresponding author, AL.

## Ethics Statement

The studies involving human participants were reviewed and approved by the Norwegian Regional Committee for Medical and Health Research Ethics, RECWest [IRB #3 (FWA00009490, IRB00001872)]. The patients/participants provided their written informed consent to participate in this study.

## Author Contributions

AL was responsible for the design and analyses, interpretation of results, and drafting the manuscript. DJ organized the data and commented on the design, results, and drafts of the manuscripts. JH contributed substantially as the head of the adult ADHD project and commented on the design, results, and drafts of the manuscript.

## Conflict of Interest

The authors declare that the research was conducted in the absence of any commercial or financial relationships that could be construed as a potential conflict of interest.

## Footnotes

1 http://www.uib.no/kgj-npd
